# Consequences and coping strategies of nurses and registered nurses perceiving to work in an environment characterized by workplace bullying

**DOI:** 10.1016/j.dialog.2024.100174

**Published:** 2024-02-24

**Authors:** Daniela Acquadro Maran, Gianmarco Giacomini, Alessandro Scacchi, Roberta Bigarella, Nicola Magnavita, Maria Michela Gianino

**Affiliations:** a Department of Psychology, Università di Torino, Torino, Italy; b Department of Sciences of Public Health and Pediatrics, University of Turin, Torino, Italy; c Department of Psychology, University of Turin, Torino, Italy; d Department of Woman and Child Health and Public Health, Fondazione Policlinico Universitario Agostino Gemelli IRCCS, Rome, Italy; e Department of Sciences of Public Health and Pediatrics, University of Turin, Piazza Polonia, 94, Torino (I) 10126

**Keywords:** Bullying in workplace, Occupational risk, Witness, Well-being, Coping strategies

## Abstract

**Aim:**

The aim of this study was to analyze the well-being and coping strategies of nurses working in an organizational setting perceived as characterized by workplace bullying. The innovative aspect of this study is that we considered only those who perceive to work in an organizational environment characterized by workplace bullying, and not those who see themselves as victims and those who perceive they work in an organizational environment not characterized by workplace bullying.

**Method:**

A questionnaire with the NAQ-R, PGWBI, Val.Mob. and Brief COPE scales was administered to nurses. To better understand this phenomenon, a comparison was made between 331 nurses and 166 workers in other professions who also work in an organizational environment perceived to be characterized by workplace bullying.

**Results:**

In both groups (nurses and workers), the results were approximately the same in terms of personal bullying and workplace bullying episodes and the number of physical and emotive symptoms. The PGWBI score was lower for nurses than for workers in other fields. Among the individual symptoms, nurses and registered nurses were more likely to report gastritis, insomnia and heartburn than workers in other contexts. Workers in other contexts were more likely than nurses to report symptoms of anxiety, fear, feelings of insecurity, inferiority and guilt. In terms of coping strategies, nurses were more likely than other workers to report distraction, substance use, emotional support, disengagement, venting, positive reframing, humor, and religion. Workers in other professional context were more likely than nurses to report active coping, denial, instrumental support, planning, acceptance, and self-blame.

**Conclusion:**

Results suggest that the consequences of working in a perceived organizational environment characterized by workplace bullying are similar for both groups of workers, with nonstatistical differences in perceived workplace bullying episodes and sum of physical and emotive symptoms.

**Implication:**

Overall, findings suggest that workplace bullying prevention is a fundamental element in training workers in all types of workplaces and should be an integral part of curriculum activities.

## 1 Introduction

Workplace bullying (WPB) has been defined by Cowie et al. [1], see also Al Omar et al. [2] as negative behavior between peers or between supervisors and hierarchically lower superiors in which the person is harassed and attacked, directly or indirectly, by one or more persons, systematically and over an extended period of time, with the purpose and/or effect of making the person feel alone. Research suggests that a significant number of nurses experience persistent WPB [3,4]. Results from the EWCTS survey [5] show that in Europe health care workers reported the highest prevalence of any type of intimidation, including WPB, compared to other sectors. In the OSH Pulse survey, healthcare workers were more likely than workers in other sectors to report experiencing violence and verbal abuse (30% vs. 16%), not only by third parties but also by colleagues (10% vs. 7%) [6]. Furthermore, a meta-analysis by Liu et al. [7] found that across the globe 61.9% of healthcare workers have experienced violence in the workplace. Factors that increase the prevalence of this risk include high workload, pressure on workers, high stress levels, and understaffing [8]. In Italy, the most reliable estimates for the prevalence of the phenomenon in the health sector are 12%, while in the commercial sector, for example, the percentage is 9% [9]. Research suggests a prevalence of the phenomenon of 10-50% in this profession [10], in contrast to other, less affected sectors such as agriculture, where a prevalence of 3% has been found [11]. In the present study, we compared nurses working in a healthcare facility and workers from other professions.  Italian healthcare facilities are organised as public institutions at local level and have a hierarchical structure with a General Manager at the top. The General Manager is responsible for the management of health services, the planning and monitoring of activities and coordinates the various operational units, such as health departments, territorial services and hospitals. He is followed in the hierarchy by the department heads (heads of the individual departments and their activities), doctors and nurses (who provide direct care to patients) and auxiliary staff (who support the activities of the healthcare company) https://www.salute.gov.it/portale/ministro/p4_5_2_6_1.jsp?label=cenniStorici&menu=cenniStorici&id=546. The aim is to understand whether the consequences and coping strategies were the same or greater. The results may be useful for the management of healthcare facilities to better understand the importance of intervention measures and the need to allocate economic resources to prevention in the healthcare sector, which is under great stress, especially in the pandemic period.

## 2 Background - WPB in Health Care Facilities

Healthcare workers are at risk of exposure to violence and bullying by third parties (i.e., patients, patients’ family members, or other bystanders) due to frustration, anxiety, pain, psychiatric factors by patients or their caregivers who may perceive a lack of attention or care [2]. The main causes of WPB in the health care sector seem to be the extremely precarious organizational conditions and complex hierarchical systems. Healthcare is an organizational place where the hierarchical structure favors constraints, one of the elements that could determine WPB behavior [12]. The predominant experience under conditions of organizational constraints is that of being forced into conditions that do not correspond to the regular performance of one's job function. The working conditions of nurses are paradigmatic of these situations: in the health care facilities, they are subject to two different hierarchies, medical and nursing, which do not always have a favorable relationship and make uniform decisions [13]. In addition, it is important to consider that nurses' attention to human needs is not always reciprocated by positive behaviors on the part of patients and their caregivers.

It should be noted that WPB is a social phenomenon in which the protagonists are the bully and the victim; however, these two people rarely face each other alone, but often act as an audience [14]. The bully may have different reasons for his actions: the fear of losing the job or the hard-earned position or being unfairly displaced by someone younger or more qualified, the fear of career that leads to overcome any obstacle that comes in one's way, the simple antipathy or intolerance towards someone with whom one has to live most of the day, etc. [15]. As described by Zapf et al. [16], the typical characteristic of the victim is isolation. There also seem to be characteristics that favor the identification of the victim in an organizational context, such as being newly hired, being "different" (the only woman in a man's office or vice versa), or still having a lot of success [16]. Finally, bystanders are all those people who are part of the work environment but are not directly involved in the WPB. Nevertheless, they play a crucial role in the further development of the phenomenon: with their intervention (or non-intervention) they can reinforce or inhibit WPB [17].

### 2.1 Consequences of WPB

People who experienced WPB are in a state of extreme distress, with feelings of powerlessness and chronic discomfort, which negatively impacts their emotional stability and sometimes affects physical and mental health [12]. In addition, the phenomenon can undermine teamwork and the ability to develop a culture of safety [18]. A number of studies have examined the negative consequences for nurses and registered nurses, finding high rates of depression and anxiety. In some cases, victims have been observed to suffer from post-traumatic stress symptoms. The most common psychosomatic symptoms are gastrointestinal complaints and symptoms of nervousness, especially frequent palpitations [19]. From a psychological perspective, it is confirmed that 50% of nurses and registered nurses who are victims of bullying show depressive symptoms [20,21] and 25% show symptoms of PTSD, especially if they have experienced WPB and sexual harassment [22]. Finally, at the social level, the victim tends to isolate and leave the workplace in the long term, even if he or she has no real chance of finding a new job in a different structure [23]. In addition, findings suggest an increase in the use of substances such as tobacco and alcohol [22]. Some previous research suggests that the phenomenon also has a negative impact on the health of those workers who are in an environment perceived to be characterized by WPB, even if the intensity of the negative behaviors is perceived to be low [24]. Furthermore, these behaviors have been shown to negatively affect not only nurses' perceived well-being, but also their performance and patient care, and in particular to increase the perceived risk to patients [25].

For the organization, the impact of WPB in healthcare can be significant, not only in terms of nurses and registered nurses' psychological well-being, but also in terms of reduced productivity and performance, loss of problem-solving skills, deterioration of the work climate, and deterioration in the quality of relationships among caregivers. There may also be high absenteeism and turnover. In addition, due to the stress experienced by those involved, as described above workers are at higher risk for errors, resulting in poor concentration and inattention [26], and they are less satisfied with their work and therefore less motivated [20].

As Nielsen et al. [27,28] note, it is important to point out that some research has shown that all workers, not just WPB targets, are victims of the phenomenon. For example, previous studies have found a large overlap between witnessing and victimization (see for example [29]), suggesting that many workers perceive themselves as potential targets of bullying. Thus, exposure to WPB may be a significant confounding factor that could explain the consequences expressed by workers that are not the main target of the WPB. A prospective study from the United Kingdom that controlled for witness’ own exposure to bullying found that in presence of WPB workers experienced work-related depression and anxiety [30]. In the longitudinal study by Holm and colleagues [31], the results show that a working environment in which bullying occurs can have a detrimental effect on the perceived quality of patient care. In a cross-sectional study of the associations between WPB and workers’ attitudes and well-being, witnessing WPB was found to be related to work-related attitudes such as intention to leave, but not to stressful experiences such as worry and need for rest, even when controlling for workers' own WPB experiences [32].

### 2.2 Coping strategies

Workers who experienced an environment characterized by WPB interpret and respond to the experience in different ways. Coping is characterized by a set of behavioral responses, exhibited by individuals facing stressful situations, which enables them to modify the surrounding environment and adapt to the stress-causing agent, with the intent of reducing discomfort. In this sense, it can be stated that coping allows reducing negative reactions to a given situation [33], thus acting as a defense mechanism, or, more specifically, as a stable and unconscious mental process used to manage internal and/or external conflicts. Studies indicate high levels of distrust in organisational procedures, which leads to unreported the misconducts (see [34,35]). Joao and Portelada [33] found that among the actions taken by nurses who suffered from workplace bullying (n = 679), the following were most frequently mentioned: telling colleagues (34.2%) what happened, confronting the perpetrator (33.1%), and ignoring the perpetrator (25.1%). 12.6% of nurses stated that they were unable to do anything about the perpetrator or did not feel able to do anything about it. A study by Karatuna and Gok [36] in healthcare sector reported that more than 50% of individuals who suffered violence do not even attempt to file a complaint of WPB because they are concerned about the potential impact on work and making the situation worse. Hampton et al. [37] found that among nurses who experienced bullying, decisive talk, leaving the organisation, and avoidance were the most commonly cited strategies to respond to bullying. Not only the direct victims of WPB, but also those who work in a hostile climate characterized by the phenomenon use defensive strategies. As suggested by Lucena et al. [38], in the health care sector, workers in environment characterized by WPB are willing to accept the problem and suggest that victims ignore the problem (downplaying and denying the misconduct) or make a positive break (e.g., behaving differently in the workplace). These findings were confirmed by Wunnenberg [35] and Hong et al. [39]. In the study conducted by Wunnenberg [36], the results showed that nurses in a WPB context use a variety of coping strategies, including trying to solve the problem, seeking social support (e.g. talking to others and seeking advice from family members) and avoidance strategies, such as disengagement. The results of the study by Hong et al. [39] show that employees who are not the target of WPB use both adaptive coping strategies (e.g. emotional support) and maladaptive strategies (e.g. venting).

## 3 The study

Considering that the risk of bullying in healthcare is higher than in other work environments and that there is sufficient knowledge about the consequences among victims, we wondered whether those who work in organizational environments perceived as WPB also experience negative consequences and what coping strategies they use. To this end, we used a questionnaire to identify individuals who perceive they work in a WPB environment but are not victims.

In order to better describe the phenomenon, a comparison was made between nurses / registered nurses and workers from other sectors. To identify the different types of participants in the study, the following terms are used below: nurses and registered nurses who perceive working in an environment characterized by WPB = NUR_WPB_; workers in other workplace, not healthcare sector, who perceive working in an environment characterized by WPB = OTH_WPB_. Based on the literature analyzed, our hypothesis is the following:

- NUR_WPB_ experience more consequences (symptomatology) than OTH_WPB_ (hp. 1a), and NUR_WPB_ experience greater consequences (in terms of higher symptomatology and less perceived well-being) than OTH_WPB_ (hp. 1b)
- NUR_WPB_ use both adaptative and maladaptative coping strategies however NUR_WPB_ are more prone to use maladaptative coping strategies than OTH_WPB_ (hp. 2b)

The research was conducted in a north-western Italian region. As suggested by Hoprekstad et al. [40], the personal history of employees who have been victims of bullying (e.g. victims of bullying at school) may influence their current perception and experience of workplace bullying. For this reason, we chose to study the phenomenon among those who perceived to work in a context characterized by the phenomenon but who do not see themselves as victims. For NUR_WPB_, the criteria for inclusion in this study were that participants had worked in the health facilities, were between 18 and 65 years old and perceive to work in an organizational environment characterized by WPB. Accordingly, exclusion criteria were age (under 18 years, over 65 years), time worked (less than six months), do not perceive to work in an organizational environment characterized by WPB and perceive to be victim of WPB.

For OTH_WPB_, the criteria for inclusion in this study were that participants had been employed in a non-healthcare, were between 18 and 65 years old and perceive to work in an organizational environment characterized by WPB. The exclusion criteria were age (under 18, over 65), time worked (under six months), working in the healthcare sector, do not perceive to work in an organizational environment characterized by WPB and perceive to be victim of WPB. The survey was conducted in 2022, during to the pandemic: in that period, the number of monthly assaults on medical staff in Italy rose from 13.5 to 27.2 [41]. This could be due to the increased number of patients, which led to stress among staff.

## 4 Method

An online questionnaire was developed to assess the WPB perceived, consequences (symptomatology and perceived well-being), and coping strategies. The question included the definition of the phenomenon. The questionnaire consists of four sections:

1. To assess the status of workers who perceive working in an environment characterized by WPB and who are not victims of WPB, the Italian version of the Negative Acts Questionnaire Revised (NAQ-R) was used [42]. The items follow an operational approach, asking participants to indicate how often they experienced various potential bullying behaviours or negative actions. The questionnaire distinguishes between personal bullying, i.e., hostile actions toward a person (PB; e.g., "spreading gossip and rumours about you”, score range 12-60), and work-related bullying, i.e., behaviours related to the work of the person who is the target of the bullying (WRB; e.g., "someone withholds information that affects your performance", score range 5-25). The response format is 1 (never) to 5 (everyday). In line with Notelaers and Einarsen [43], participants with a total score of more than 33 were classified as victims of bullying (see also [44]) and were therefore excluded from this study. At the same time, respondents who answered "never" to all questions were classified as not perceiving to work in an environment characterized by WPB (PB = 12 and WRB = 5). These participants were also excluded. We therefore included in the study participants with a score above 17 and below 33. In this study, Cronbach's alpha = .90.

2. The Val.Mob. Symptomatology Scale [45] was used to assess participants' health status. The scale consists of 23 items (e.g., insomnia, anxiety symptoms) with response options ranging from 1 = never to 5 = always. The items from the Val.Mob. Symptomatology Scale were analyzed both as single symptom (score range for each symptom 1-5) and sum of symptoms (total score range 23-115). (Cronbach's alpha = .94)

3. To assess perceived quality of life and psychological well-being was used the Italian version of the Psychological General Well Being Index (PGWBI) [46]. The PGWBI questionnaire, which comprises 22 items, makes it possible to measure the level of stress based on the self-perceived rating [46]. The questions cover six aspects: anxiety, depressed mood, positive well-being, self-control, general health and vitality. Each scale comprises 3–5 items. The questions allow multiple-choice answers with scores between 0 and 5 (best score). The PGWBI total score is the sum of all items and ranges from 0 to 110. Higher scores indicate greater psychological well-being. (Cronbach's alpha = .70)

4. The Brief COPE scale [47,48] was used to assess coping strategies. Each strategy corresponds to a series of questions asked in random order; the strategy with the highest score (from 2 to 8) is the most frequently used. It should be noted that the use of adaptive, i.e. constructive and functional, or, on the contrary, maladaptive strategies is considered an indicator of the degree of psychological well-being or discomfort. The maladaptive coping strategies are the following: venting, denial, substance use, behavioural disengagement, self-blame. The adaptative coping strategies are the following: active coping, positive reframing, planning, humour, acceptance, emotional support, religion, instrumental support. (Cronbach's alpha = .96).

### 4.1 Procedure

A letter was sent to two health facilities in cities in the north-western part of the country explaining the purpose of the survey and mentioning privacy and anonymity. These health facilities were selected because both are organizations that operate territorial services and a hospital in a non-metropolitan area. They are a typical example of health facilities in this region of the country. A meeting was held to better explain the purpose of the survey and the process. A formal communication within the organization included the description of the project and the link to the questionnaire. At the same time, staff from other organizations (outside of healthcare) were snowballed. Each participant was asked to send the link to the questionnaire to five friends/colleagues. The link was active during the period from January to May 2022.

### 4.2 Participants

The online questionnaire was emailed to 1650 (80% female, 20% male; mean age 49.5 years, range = 22-67 years) nurses and 1200 workers in other workplaces. The questionnaire was completed by 581 individuals (346 nurses, 21%; 194 workers in another context, 16.2%). Based on the consideration that the total number of nurses was 1650 and we did not know in advance the percentage of people who could agree to participate in the survey, we assumed a prevalence of 50%. The minimum number of observations to achieve a confidence level of 95% and a margin of error of 5% was 312. The 346 responses we collected left enough margin. For workers in a different context, we assumed a prevalence of 30%, in line with Manfreda et al. [49,50], who found that the average response rate to online surveys was 11% and the 95% confidence interval was 6–15%. The minimum number of observations to achieve a 95% confidence level and a 5% margin of error was 134, so the 194 responses were considered sufficient. Calculations were performed using Calculator.net (https://www.calculator.net/sample-size-calculator (accessed December 15, 2021)). There were no missing data as online survey software required participants to answer all questions. A total of 84 of them were excluded because they did not meet the criteria for inclusion in the study: 12 had a NAQ-R score ≥ 34 (all nurses and registered nurses), 44 nurses and registered nursed and 27 workers in other organization had a NAQ-R score = 17 (PB = 12 and WPB = 5). One worker in other organization was excluded because he was over 65 years old. The participants that perceive to work in an environment characterized by WPB were 497 (85.5%), most part (59.5%) were female. Six indicated ‘not-binary gender’. They were on average 41.10 years old (range = 18-65; s.d. = 14.24) and had worked 14.77 years (range = 6 months-40 years; s.d. = 13.44). The majority were married (260, 52.4%), 139 (28%) were engaged, 73 (14.7%) were single and 12 (2.4%) were divorced. Twelve participants didn’t give an answer (they have chosen the following option: "I do not want to indicate my marital status").

### 4.3 Data analysis

Data were processed using SPSS version 28 (IBM Corp., Armonk, NY, USA). Descriptive frequency analyses were calculated for categorical variables, χ^2^ tests were used to measure differences between groups. Descriptive analyses of mean and standard deviation were calculated for numerical variables. T-tests were used to examine differences between the two groups (NUR_WPB_ and OTH_WPB_); results were considered statistically significant when p <.05. A correlation was performed to relate perceived psychological well-being (PGWBI), experiences of personal (PB) and work-related bullying (WRB), symptomatology, and coping strategies.

### 4.4 Ethical considerations

This study conformed to the provisions of the 1995 Declaration of Helsinki, as revised at the 2000 Edinburgh meeting [51]. All relevant ethical guidelines were followed, including compliance with Italian legislation. The research project was approved by the Ethics Committee of the XXX before the start of the study (no. 0654314-07/12/21). Because there was no medical treatment or other procedure that could cause biological, psychological, or social harm to the participants involved, no additional ethical approval was required. Participation was voluntary, and no one received compensation for their participation.

## 5 Findings

Of the total participants, 331 are NUR_WPB_ and 166 are OTH_WPB_ who have NAQ scores between 18 and 33. In Table 1 there are the socio-demographic characteristics of the participants.

**Table 1:** Socio-demographic characteristics of the participants (N = 497)

	NUR_WPB_ (n = 331)	OTH_WPB_ (n = 166)
Age	47.94	33.12
	(s.d = 10.15)	(s.d.= 14.19)
Sex:		
Female	85.4%	29.5%
Male	12%	69.9%
Non-binary	2.6%	0.6%
Working year	19.59	8.23
	(s.d. = 12.99)	(s.d.= 11.08)

As shown in Table 1, the mean age and the work experience of OTH_WPB_ participants is lower than NUR_WPB_ participants (respectively t = 11.09, p = .001 and t = 8.56, p = .001). There is also a difference between genders, with the majority of NUR_WPB_ being women and the majority of OTH_WPB_ being men (χ^2^ = 125.75, p = .001).

Table 2 shows the results of the NAQ-R, PGWBI, Val.Mob. symptoms and coping strategies in NUR_WPB_ and OTH_WPB_.

**Table 2:** Personal Bullying, Work-related Bullying, PGWBI, symptoms and coping strategies scores (N = 497)

	NUR_WPB_ (n = 331)	OTH_WPB_ (n = 166)	*t*	*p*
PB	17.73(7.46)	17.54(5.70)	0.28	n.s.
WRB	9.55(3.59)	9.17(2.89)	1.12	n.s.
PGWBI	20.37(4.63)	22.48(2.93)	-5.10	.001
Symptoms:	47.22(18.64)	49.50(17.98)	-1.21	n.s.
Gastritis	2.10(1.25)	1.89(1.08)	2.49	.013
Headache	2.46(1.23)	2.59(1.17)	-1.06	n.s.
Anxiety symptoms	2.34(1.28)	2.80(1.25)	-3.50	.001
Demoralization	2.69(1.28)	2.75(1.16)	-0.53	n.s.
Nausea	1.49(0.87)	1.49(0.89)	-0.01	n.s.
Insomnia	2.49(1.36)	2.20(1.22)	2.24	.028
Crying crisis	1.76(1.01)	1.90(1.16)	-1.27	n.s.
Heartburn	2.23(1.28)	1.88(1.13)	2.86	.005
Tachycardia	2.12(1.30)	1.94(1.15)	1.39	n.s.
Panic attacks	1.47(0.99)	1.55(1.02)	-0.75	n.s.
Muscle tension	2.47(1.33)	2.34(1.27)	0.99	n.s.
Nervousness	2.89(1.31)	3.11(1.20)	-1.69	n.s.
Sadness	1.83(1.17)	1.89(1.25)	-0.50	n.s.
Aggressiveness	2.07(1.26)	2.20(1.20)	-1.00	n.s.
Apathy	2.04(1.19)	2.10(1.14)	-0.48	n.s.
Fears	1.68(1.10)	2.08(1.29)	-3.26	.001
Relationship difficulties	1.75(0.99)	1.88(1.10)	-1.19	n.s.
Feeling of insecurity	1.73(1.09)	2.36(1.30)	-5.05	.001
Feelings of inferiority	1.72(1.05)	2.29(1.36)	-4.50	.001
Problems with memory	2.24(1.25)	2.23(1.24)	0.12	n.s.
Feelings of guilt	1.75(1.07)	1.98(1.23)	-1.98	.048
Attention problems	2.07(1.21)	2.19(1.13)	-0.99	n.s.
Thoughts of critical events at work	1.83(1.15)	1.90(1.16)	-0.53	n.s.
Coping strategies:				
Self-Distraction	8.15(1.65)	5.22(1.62)	5.43	.001
Active-Coping	2.99(1.41)	5.94(1.71)	-18.09	.001
Denial	2.18(0.71)	3.01(1.33)	-7.74	.001
Substance Use	4.78(1.71)	2.47(1.32)	14.27	.001
Emotional Support	5.14(1.59)	4.70(1.79)	2.46	.014
Instrumental Support	3.21(1.40)	5.16(1.81)	-11.67	.001
Disengagement	4.94(1.44)	3.31(1.41)	10.97	.001
Venting	5.58(1.76)	4.67(1.51)	5.22	.001
Positive Reframing	6.26(1.60)	5.31(1.64)	5.63	.001
Planning	4.09(1.61)	6.15(1.53)	-12.54	.001
Humour	5.90(1.59)	4.18(1.47)	10.71	.001
Acceptance	4.03(2.13)	5.74(1.45)	-9.13	.001
Religion	5.34(1.53)	3.36(1.84)	11.11	.001
Self-Blame	5.73(2.70)	6.70(2.37)	-3.73	.001

Table notes. *t* = t-test value; *p* = p value; n.s. = not statistical significance.

As shown, NUR_WPB_ and OTH_WPB_ showed approximately equal scores on PB, WRB, and number of symptoms. The PGWBI score was lower in NUR_WPB_ than OTH_WPB_. For individual symptom, NUR_WPB_ were more prone to indicate gastritis, insomnia, and heartburn than OTH_WPB_. OTH_WPB_ were more likely than NUR_WPB_ to report anxiety symptoms, fear, feelings of insecurity, feelings of inferiority and guilty. In terms of coping strategies, NUR_WPB_ were more likely than OTH_WPB_ to report self-distraction, substance use, emotional support, disengagement, venting, positive reframing, humour and religion. OTH_WPB_ were more likely than NUR_WPB_ to report active coping, denial, instrumental support, planning, acceptance and self-blame.

Table 3 shows the results of correlating PB and WRB episodes with perceived well-being, symptoms and coping strategies.

**Table 3:** Correlation analysis (N = 497)

	NUR_WPB_ (n = 331)		OTH_WPB_ (n = 166)	
	PB	WRB	PB	WRB
PGWBI	0.01	0.11	-0.50**	-0.34**
Symptoms	0.52**	0.51**	0.56**	0.49**
Coping strategies:				
Self-Distraction	-0.02	-0.19**	0.14	0.17*
Active-Coping	0.24**	0.16*	0.08	0.17*
Denial	0.06	0.17*	0.12	0.25**
Substance Use	-0.02	-0.06	0.24**	0.14
Emotional Support	-0.04	-0.11	0.09	0.03
Instrumental Support	0.31**	0.19**	0.08	0.00
Disengagement	0.12	0.08	0.26**	0.19*
Venting	-0.11	0.17*	0.16*	0.23**
Positive Reframing	-0.11	0.14*	0.05	0.11
Planning	-0.08	-0.02	0.03	0.15
Humour	-0.07	-0.07	-0.01	0.05
Acceptance	-0.06	-0.05	0.03	-0.01
Religion	0.06	-0.03	0.10	0.14
Self-Blame	0.09	0.08	0.03	0.05

Table notes. * *p* < 0.05; ** *p* < 0.01

As shown in Table 3, for NUR_WPB_, experienced symptoms increased when perceived WPB (both PB and WRB) increased in their organization. In addition, increasing experience of PB episodes leads to an increase in the use of coping strategies such as active coping and instrumental support. With increasing experience of WRB, self-distraction decreased, while instrumental support, venting, and positive reframing coping strategies increased. and disengagement and a decrease in positive reframing and planning. For OTH_WPB_, an increase in PB and WRB episodes resulted in a decrease in perceived well-being and an increase in symptoms. An increase in PB and WRB episodes led to an increase in disengagement and venting coping strategies. Increases in PB episodes lead to increases in substance use, whereas increases in WRB episodes lead to increases in the following coping strategies: self-distraction, active coping, and denial.

## 6 Discussion

The aim of this study was to analyze the well-being and coping strategies of nurses perceiving to work in an organizational setting characterized by WPB. To better understand this phenomenon, a comparison was made with workers in other professions who also work in an organizational environment characterized by WPB. The innovative aspect of this work is that we considered only those who perceive an organizational environment characterized by WPB, and not those who see themselves as victims and those who perceive they work in an organizational environment not characterized by WPB. In both groups (nurses and registered nurses and workers), the variables of perceived well-being, symptoms, and coping strategies used were examined. Results suggest that the consequences of working in a perceived organizational environment characterized by WPB are similar for NUR_WPB_ and OTH_WPB_, with non-statistical differences in those who perceived working (or not) in an environment characterized by WPB (both PB and WRB) and the sum of symptoms. Therefore, hypothesis 1a was not confirmed: NUR_WPB_ do not experience more consequences (symptomatology) than OTH_WPB_. A statistical difference was found in perceived well-being: NUR_WPB_ more often reported a lower score than OTH_WPB_. In addition, symptoms reported by NUR_WPB_ were more often physical (gastritis, heartburn, insomnia) than by OTH_WPB_, whereas OTH_WPB_ reported more emotional symptoms (fear, feelings of insecurity, feelings of inferiority, and guilt) than NUR_WPB_. Thus, hypothesis 1b was partially confirmed: NUR_WPB_ have lower perceived well-being but not more symptoms than OTH_WPB_ and both in NUR_WPB_ and OTH_WPB_ the correlational analysis, results show that PB and WRB episodes increase the negative symptoms. From the comparison between NUR_WPB_ and OTH_WPB_ of the individual symptoms scores, appears that NUR_WPB_ are more likely to express their discomfort through physical symptoms. As suggested by Gullander et al. [52], these are stress symptoms that occur in individuals who perceived working in an environment characterized by WPB, especially when the stress is prolonged. It is important to note that we did not examine whether participants described themselves as victims or witnesses: we used the NAQ-R to identify workers who found themselves in an environmental organization characterized by WPB. As suggested by Nielsen and Einarsen [53], psychological distress should be a predictor of perceived WPB, not an outcome. Thus, workers who perceive they work in an organizational environment characterized by WPB may be sensitive to the phenomenon and may perceive some behaviors, such as rudeness and unkindness, as violence that actually affects a target person but could be directed against them in the future. Consistent with the gloomy perception mechanism [54], workers’ descriptions of their organizational environment and the consequences they experience may be influenced by a negative perception bias, and prevalence rates of perceived WPB episodes may be influenced due to this type of bias. Future research could analyze perception bias to better understand the extent to which perceptions of working in an organizational environment characterized by WPB are related to sensitivity to the phenomenon, perhaps because participants have had previous experiences of victimization (for this or other forms of violence, such as bullying at school or domestic violence). Another statistically significant difference between NUR_WPB_ and OTH_WPB_ was the coping strategies used. NUR_WPB_ were more likely than OTH_WPB_ to report both adaptive coping strategies such as emotional support, positive reframing, humor, and religion and maladaptive coping strategies such as self-distraction, substance use, and venting [55]. OTH_WPB_ were more likely than NUR_WPB_ to report self-blame and denial, which are considered maladaptive coping strategies [56], and active coping, instrumental support planning, and acceptance, which are considered adaptive coping strategies [55]. Thus, NUR_WPB_ were more likely than OTH_WPB_ to report both adaptive and maladaptive coping strategies, and NUR_WPB_ were more likely than OTH_WPB_ to report maladaptive coping strategies. Hypothesis 2 was confirmed.

In terms of coping strategies, correlational analysis showed that for NUR_WPB_, an increase in PB episodes led to an increase in coping strategies classified as adaptive, whereas the WRB episode led to both an increase and a decrease in coping strategies classified as maladaptive (see [57]). Interestingly, NUR_WPB_ reported being less self-distraction coping strategy when they perceived WRB, as if they needed to pay more attention to what was happening at work. However, it is important to note that, as suggested by AlJhani et al. [58], the use of maladaptive coping strategies is associated with the risk of burnout. Specifically, as noted by Ogus [59], in the healthcare setting, frequent use of maladaptive coping strategies in conjunction with a stressful workplace may increase the risk of burnout for nurses. In the OTH_WPB_ study, increases in PB and WRB episodes were critical for increases in maladaptive coping strategies and increases in an adaptive coping strategy, active coping, when WRB episodes increased. Active coping is a strategy that states that workers make efforts to eliminate or avoid the stressor. Thus, these strategies suggest that OTH_WPB_ are trying to cope with the phenomenon. The data collected did not allow us to understand the behaviors used to cope with the WPB episode, whether it was finding another job, changing departments within the same company, or confronting the bully and/or victim. Future research could analyze the coping strategies used using a qualitative method to further explore how workers cope with the phenomenon.

### 6.1 Limitations and future directions

This study inevitably has limitations. First, the number of respondents is small compared to the number of potential participants. One possible explanation lies in the phenomenon under study; perhaps only those who perceived they work in an environment characterized by WPB responded. In addition, the period in which we completed the questionnaire was at the end of a particularly stressful time. NUR_WPB_ experienced the pandemic firsthand, which created an unexpected workload. Their workload increased over time, and the survey was conducted at the end of one of the pandemic waves. The results obtained may therefore have been biased by fatigue, which worsened perceptions of professional relationships (between staff and with patients and caregivers). Future studies could replicate the work at a time when there is less stress. In addition, we did not examine whether increased fatigue during the pandemic worsened perceptions of the phenomenon. Future studies could use interviews to examine reports of this period to assess whether there were elements that increased the risk of victimization and to monitor the phenomenon. At the same time, the OTH_WPB_ who participated in this study were younger and had less work experience than nurses. Interestingly, the perception of both PBs and WRBs is the same as that of nurses. These data can also be related to the pandemic situation, to the difficulty of a work environment inevitably characterized by the stress caused by uncertainty and an environment perceived as hostile. Overall, the results obtained should be taken with caution and should not be generalized. Finally, respondents may have participated in order to convey a certain image of the organization. In particular, in the health care sector, where there were many attacks on the image of the organization and the profession during the pandemic, attacks due to the difficulty of managing the emergency. This is related to social desirability bias, which is the tendency of participants to give answers that they believe convey a positive image of them, and may have also influenced the results of the study [60]. Further research could explore the phenomenon of social desirability in the study of WPB in health care.

### 6.2 Implications for policy and practice

Despite these limitations, the results suggest that WPB prevention is a fundamental element in the training of workers in all types of workplaces and should be an integral part of curriculum activities. In particular, for NUR_WPB_, the experience of working in an environmental organization perceived as characterized by WBP could affect their perceived health, their ability to cope with the phenomenon and to provide professional help and care to patients and emotional and practical support to caregivers. The risk of burnout also has an impact on personal well-being and that of the organization as a whole, which is at risk of losing qualified staff.

## 7 Conclusion

The results of this study suggest that it is important to study the phenomenon of WPB not only among the victims, but also among the employees who perceive to work in an organization characterized by WPB, and that this can contribute to making the organization a safer place to work [61]. It should be noted that the first step in preventing WPB is not only to acknowledge the existence of the phenomenon, but also to ensure that the misconduct is properly reported. WPB episodes must be documented using a reporting system developed by health care providers [62]. Next steps could include implementing a WPB prevention program that includes educational, organizational, and medical interventions essential to reducing the risk of misconduct [63]. Relationship skills training could contribute to the prevention of WPB, as a lack of social skills is considered one of the precursors of the phenomenon at the individual level. At the individual level, the use of verbal self-defense or de-escalation techniques could be useful, as they allow learning how to deal with and manage everyday conflicts. Often, conflicts arise from our inability to respond to provocations. As van der Brande et al. [64] argue, coping mechanisms may play an important role in the link between the situation and its outcomes. In other words, the impact of WPB on perceived health may depend on the tendency to use certain coping mechanisms. The interaction between workers' reactivity to the context and their personal tendency to use a particular coping mechanism may thus mitigate or exacerbate the outcomes of WPB. Therefore, it is important to use adaptative coping strategies to respond appropriately, for example, to the words that are always at hand to prevent a single insult from becoming a dynamic of violence [65]. In addition, specific training on WPB can raise awareness among an organization's staff about how to characterize and respond to specific incidents [66]. At the organizational level, it may be useful to manage organizational climate by monitoring its variables in regular analyzes and making improvements [67]. In addition, explicit prevention policies (e.g., the "zero tolerance policy") that make clear which behaviors are acceptable and which are unacceptable in the workplace [68] may be useful in establishing rules that can discourage violent behavior and influence workers' perceived quality of work life.

## Funding

The authors declare that no funds, grants, or other support were received during the preparation of this manuscript.

## CRediT authorship contribution statement

**Daniela Acquadro Maran:** Writing – review & editing, Writing – original draft, Supervision, Methodology, Formal analysis, Conceptualization. **Gianmarco Giacomini:** Writing – review & editing, Project administration, Investigation, Data curation, Conceptualization. **Alessandro Scacchi:** Writing – review & editing, Project administration, Methodology, Investigation, Data curation. **Roberta Bigarella:** Writing – review & editing, Project administration, Investigation, Formal analysis, Data curation. **Nicola Magnavita:** Writing – review & editing, Writing – original draft, Methodology, Formal analysis, Data curation. **Maria Michela Gianino:** Writing – review & editing, Writing – original draft, Project administration, Investigation, Data curation, Conceptualization.

## Declaration of competing interest

The authors have no competing interests to declare that are relevant to the content of this article.

## Data Availability

The data that support the findings of this study are available from the corresponding author upon request.
